# Automated Metadata Review to Support Result Release in Quantitative Liquid Chromatography Coupled With Tandem Mass Spectrometry Applications

**DOI:** 10.1002/ansa.70111

**Published:** 2026-09-20

**Authors:** Verena Endt, Katharina Habler, Michael Vogeser

**Affiliations:** ^1^ Institute of Laboratory Medicine, LMU University Hospital, LMU Munich Munich Germany

**Keywords:** data management, LC‐MS/MS, metadata, peak review, quality assurance, result release

## Abstract

Quantitative multi‐target analysis of biological samples using isotope‐dilution liquid chromatography coupled with tandem mass spectrometry (LC‐MS/MS) is widely applied in laboratory diagnostics and life sciences now. These analyses generate a large amount of metadata (e.g. ion ratios, peak width, etc.) that must be reviewed before results can be released. Until now, these complex and time‐consuming tasks are performed mainly by technicians without dedicated software support. To explore whether practical tools for supporting result validation and release can be developed using standard spreadsheet software. A script, named MS Excel‐based evaluation macro (MSVal), was developed in Microsoft Excel. Data sets from the quantification software of an LC‐MS/MS system are imported into this tool. Validation criteria as quality assurance rules for analytical series and individual samples are defined within MSVal. The tool's functionality was evaluated using datasets from cystic fibrosis transmembrane conductance regulator (CFTR)‐modulator therapeutic drug monitoring (TDM) analytical series. After importing raw MS data, MSVal generates a list of results that meet predefined validation criteria. MSVal also produces a validation report for each analytical series. Results that do not meet validation criteria are highlighted and must be specifically addressed by the operator during the release process. MSVal was successfully tested with real‐world datasets. The development of software applications to support validation and result release in quantitative isotope‐dilution LC‐MS/MS analysis is feasible using standard software. Further refinement and progression toward commercial solutions appear valuable to close a significant gap in the clinical application of mass spectrometry.

## Graphical abstract

Quantitative isotope‐dilution liquid chromatography coupled with tandem mass spectrometry analyses generate extensive metadata that require time‐consuming manual validation in laboratory workflows. We developed *MSVal*, an Excel‐based tool that applies predefined quality criteria to automatically evaluate results and highlight deviations. This approach enables efficient validation and reporting, demonstrating that practical software solutions can enhance clinical mass spectrometry workflows.

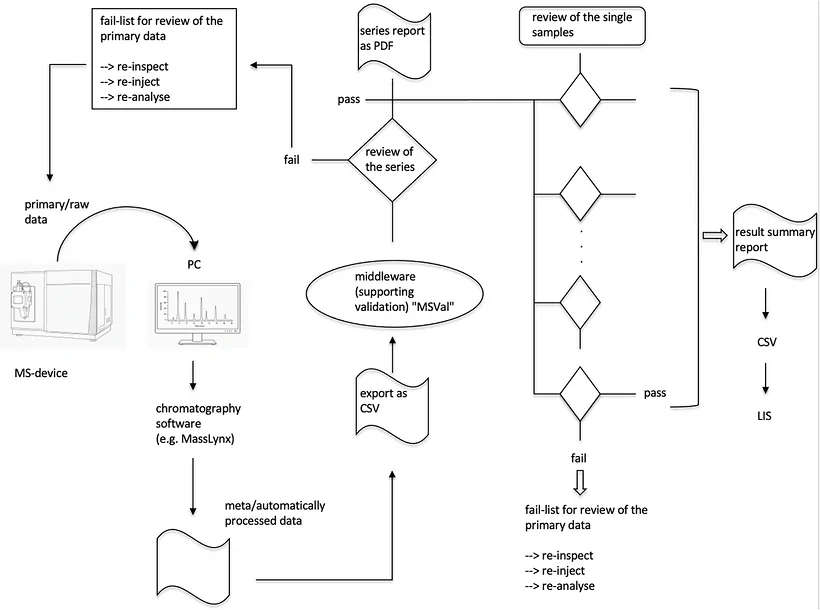

**Abbreviations**
CFTR: cystic fibrosis transmembrane conductance regulator
EU‐IVDR: European Union In vitro Diagnostic Regulation
IS: internal standard
LC‐MS/MS: liquid chromatography coupled with tandem mass spectrometry
LIS: laboratory information system
LLOQ: lower limit of quantification
MS: mass spectrometry
QC: quality control
RT: retention time
TDM: therapeutic drug monitoring.

## 1 Introduction

Liquid chromatography coupled with tandem mass spectrometry (LC‐MS/MS) with isotope dilution internal standardisation has become a significant tool in medical diagnostics, as well as in other areas of bioanalysis [1]. Although the first fully automated, calibration‐stable, random‐access analytical systems with high user‐friendliness have entered the market [2], most applications currently—and for the foreseeable future—still rely on complex analytical solutions in specialised high‐complexity medical laboratories.

In the routine operation of medical MS laboratories, post‐analytical data management is highly time‐consuming and represents a particular challenge in addition to sample preparation and instrument handling. In extensive multi‐analyte quantifications, conventional review of analytical series and individual samples with numerous multiple‐reaction‐monitoring traces for result release represents a very substantial workload for highly specialised staff. With growing sample numbers in referral laboratories, post‐analytical data management has become a bottleneck that ties up expert personnel and hampers further development in this field.

Although analytical interferences in LC‐MS/MS are likely rare, the release of series and results is a highly responsible process [3, 4]. Practices of metadata review are found to be highly heterogeneous between laboratories [5]. However, this part of the analytical process is essential for patient safety—in particular in medication management. Therapeutic drug monitoring (TDM) is a typical field of application for LC‐MS/MS in clinical laboratories and is gaining importance within the concept of precision dosing, where the highest level of analytical reliability is critical. Interference can potentially arise—among other issues—from compounds co‐eluting with the target analytes or the respective internal standards (ISs) and sharing mass transitions (isomeric/isobaric interferences) [6]; such interference can potentially be detected by suspicious peak width or ion ratios—i.e., analytical metadata. Compromised function of system components arising during an analytical run as well can potentially lead to incorrect results and may be recognised by metadata review.

Result release with peak review relies on inspection, interpretation, and assessment of a large volume of metadata (e.g. ion ratios and retention times [RTs]), which is currently carried out manually and—realistically—often involves subjective judgments. It seems therefore reasonable to apply software‐based tools for standardised metadata analysis, supporting result release, reducing human workload while maintaining the highest safety standards in result release.

Vendor‐provided chromatography‐MS software packages handle essential parts of primary data processing (peak identification, integration, constructing integration lines, calculation and partial evaluation of ion ratios, RT, etc.), but validation options in the context of entire analytical series remain insufficient. A middleware solution, positioned between MS instrument software and the laboratory information system (LIS), appears promising.

Currently, only a single commercial provider exists in this field, offering post‐analytical data analysis as cloud‐based software‐as‐a‐service [7]. However, this solution is not expected to become globally available in the near future. Thus, the authors identify a significant market gap in the application of MS in clinical laboratories, which is not readily explained. One potential explanation may be the limited size of the market.

Based on reports presented at scientific conferences, several laboratories have developed in‐house solutions for this type of workflow. However, these tools are proprietary, lack transparency, and are not publicly available for broader implementation [8, 9].

For these reasons, we decided to explore whether middleware for supporting MS metadata analysis in workflows of assisted result release can be developed in‐house using widely available standard software.

In the context of reference measurement procedures, we have already demonstrated the feasibility of such an approach [10]. The present goal was to determine whether a suitable format can also be implemented for simple and user‐friendly application in routine laboratories.

## 2 Materials and Methods

Data analysis was performed using Microsoft Excel (Version 16.101.3 for Mac, Microsoft Corporation, Redmond, WA, USA), with custom‐developed Visual Basic for Applications scripts. It can be used for both Windows and Mac operating system versions, from Microsoft Excel 2021 or Excel 365 onwards. As exemplary datasets, anonymised LC–MS/MS measurement metadata of cystic fibrosis transmembrane conductance regulator–modulator TDM (CFTR‐TDM) were used [11]. The datasets contained no patient identifiers and were non‐re‐identifiable. As only fully anonymised patient samples were used that were not obtained specifically for use in this study through an interaction or intervention with living individuals, neither informed consent nor IRB review was required.

LC–MS/MS analyses were carried out using an Xevo TQ‐XS (Waters Corporation, Milford, MA, USA), and data acquisition and quantification were performed with MassLynx and TargetLynx software (Version 4.2; Waters Corporation, Milford, MA, USA).

A catalogue of acceptance criteria was developed and implemented in an MS Excel‐based evaluation macro (referred to as *MSVal*); quantitative thresholds and deviations applied to each variable could be flexibly defined by the user.

For several criteria, reference data were generated from calibrators, quality controls (QCs), and samples within the respective series (e.g., mean RT of a compound observed in the series). In these cases, outlier testing was implemented in the calculation of series‐specific reference data.

The intended workflow was as follows: routine sample measurement and quantification were performed using the standard laboratory LC‐MS/MS procedure. Following chromatographic integration, calibration, and quantification in TargetLynx, an export file (csv format) was produced. Relevant data tables were transferred into the *MSVal* tool via copy‐and‐paste functionality, after which automated data evaluation was executed instantaneously. In a specified sheet, the tool provided: (i) results meeting all predefined acceptance criteria, and (ii) a list of samples requiring targeted peak review by an operator (since validation criteria were violated), or, where appropriate, re‐injection or full re‐analysis including sample extraction.

The following fields were imported from TargetLynx export files in the following order: line number; sample name; sample ID; type; standard concentration; result; % deviation (for calibrators and QCs); vial position; injection volume; RT; peak area; area qualifier; quantifier/qualifier ion ratio; RT of IS; IS area; response; signal‐to‐noise ratio; height/area; peak width; peak asymmetry; peak start time; peak end time; acquisition time; acquisition date.

Variables considered for the acceptance of each individual sample result are listed in Table 1. Variables used for general analysis and series‐level acceptance of analytical runs are presented in Table 2.

**TABLE 1:** Quality assurance rules for each sample.

Quantification of the target analyte in a given sample by the chromatography software in use shall be accepted if the following criteria are fulfilled:	
1)	The peak width at half height lies within the predefined acceptance range (± x%) relative to the median of the peak widths at half height observed for the calibrators in the analytical run.^*^
2)	The peak asymmetry lies within the predefined acceptance range (± x%) relative to the median of the peak asymmetry observed for the calibrators in the analytical run.*
3)	The ion ratio lies within the predefined acceptance range (± x%) relative to the median of the ion ratio observed for the calibrators in the analytical run.*
4)	The peak start time/end time lies within the predefined acceptance range (± x%) relative to the median of the peak start time/end time observed for the calibrators in the analytical run.^*^
5)	The retention time lies within the predefined acceptance range (± x%) relative to the median retention time of the internal standard observed for the analytical series.^*^
6)	The peak area of the internal standard lies within the predefined acceptance range (± x%) relative to the median peak area of the internal standard observed for the calibrators and QCs in the analytical run.
7)	The retention time of the internal standard lies within the predefined acceptance range (± x%) relative to the median retention time of the internal standard observed for the calibrators and QCs in the analytical run.
8)	The relative retention time of the analyte lies within the predefined acceptance range (± x%) relative to the relative retention time observed for calibrators and QCs (relative retention time = retention time analyte:retention time IS).^*^

* If deviations exceed limits, the software shall flag the sample result as requiring particular review by the operator.

**TABLE 2:** Quality assurance rules for general analysis and analytical series.

An analytical batch (as processed by the respective chromatography software) is accepted if all of the following criteria are fulfilled:	
1)	**Calibration accuracy (back‐calculation)**: The deviations between the back‐calculated concentrations of the calibrators and their nominal concentrations are within a predefined acceptance range (± x%).
2)	**QC performance**: The deviations of the quality control (QC) sample results are within their respective predefined acceptance limits (± x%).
3)	**Carry‐over**: No carry‐over is observed for either the internal standard (IS) or the target analyte, according to predefined specifications (± x%).
4)	**Internal standard retention time precision**: The coefficient of variation (CV) and relative deviation of the internal standard retention times across the batch remain below a predefined threshold (± x%).
5)	**Internal standard retention time stability (trend analysis)**: The internal standard retention time remains stable throughout the batch, within a predefined range (± x% between the first and the last third injections), including outlier tests.
6)	**Internal standard signal precision**: The coefficient of variation (CV) and relative deviation of the internal standard peak area across the batch remain below a predefined threshold (± x%).
7)	**Internal standard signal drift (trend analysis)**: The drift of the internal standard peak area throughout the batch does not exceed the predefined acceptance limit (± x% between the first and last third injections), including outlier tests.
8)	**Matrix effect monitoring (IS area vs. solvent)**: The median internal standard peak area of the batch does not deviate by more than the predefined limit (± x%) from the IS peak area measured in a solvent injection within the same batch.

The tool was evaluated in parallel with conventional (manual) data review in routine practice using 10 analytical series.

## 3 Results and Discussion

**FIGURE 1:**
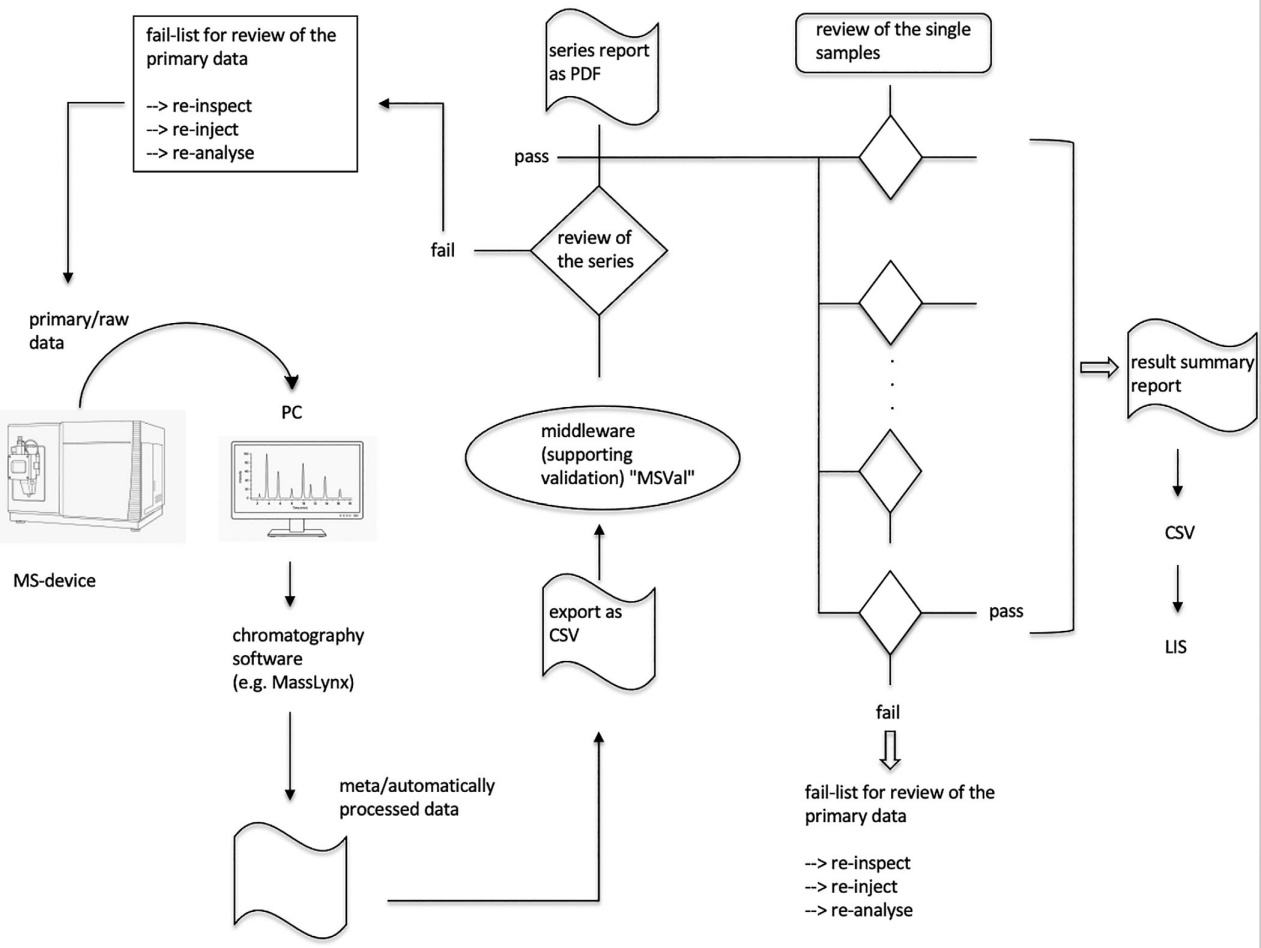
Scheme of the workflow of the automated metadata review of *MSVal*.

We present here an Excel‐macro solution (designated *MSVal*) designed to support the safe and efficient release of MS results in biomedical laboratories, demonstrating the feasibility of this approach. Although similar in‐house tools exist, no solution has been described in detail in the published literature to date. The scheme of *MSVal* is shown in Figure 1.

The tool *MSVal* is available as Supporting Information with this publication. The pre‐entered data and acceptance criteria are provided for illustrative purposes only and should be replaced or adapted according to the specific assay and laboratory requirements. It is not approved for use in diagnostic procedures; it is intended solely to help readers test the principle and the rules using their own sample data.

The implementation consists of an Excel file with macros and multiple worksheets:

The first worksheet provides instructions for use (‘User Instructions’).

The second worksheet is used for data import (Excel tables exported from TargetLynx; ‘Input’).

The third worksheet presents the summary of results with samples that are accepted and not accepted according to the user‐defined acceptance criteria (‘Output’): (i) listing of sample results that require further action (in particular, targeted review by the operator or a supervisor/senior operator as the next step), (ii) listing of sample results that meet the (user‐defined) acceptance criteria for result release, and (iii) evaluation of the analytical run.

The fourth worksheet allows for entering acceptance criteria (‘Criteria Input’).

Additional worksheets provide visualisation of intermediate results (‘General Analysis’, ‘Analysis of each sample’ and ‘Series Analysis’).

*MSVal* can export reports both as PDF files and as result lists in CSV format for transfer to a Laboratory Information System (LIS).

The suggested workflow follows a ‘review by exception’ or ‘accept/reject’ approach. Primary peak detection, integration, and quantification are performed by the MS software (e.g. MassLynx), while *MSVal* provides a structured metadata‐based quality assessment of the run and individual samples, respectively. Any flags raised by *MSVal* trigger an escalation step, prompting review by a senior analyst. Co‐eluting peaks or incorrect peak detection by the primary chromatographic software are expected to be detected with high probability through deviations in peak width, peak‐start and ‐end‐time and peak asymmetry in particular. Possible corrective actions include re‐integration, re‐injection, or entire re‐processing of the affected sample, each tracked by appropriate internal documentation.

The performance and practical applicability of *MSVal* were evaluated using 10 analytical runs from our CFTR TDM workflow. The dataset comprised the seven CFTR‐modulators included in the study, namely ivacaftor, ivacaftor‐M1, ivacaftor‐M6, tezacaftor, tezacaftor‐M1, elexacaftor and elexacaftor‐M23. For each analytical run, six calibrators, approximately 12 QCs and around 30 patient samples were assessed according to our predefined method‐ and analyte‐specific acceptance criteria. Overall, the datasets comprised approximately 2100 (10×7×30) patient sample results, 840 (10×7×12) QC results, and 420 (10×7×6) calibration results across the 10 analytical runs.

For each run, the conventional workflow, including manual metadata review and result release, was compared with *MSVal*‐assisted review. Both workflows were performed by experienced operators, and the time required for the review was recorded for both approaches. The conventional review required approximately 30 min per analytical run on average, whereas *MSVal*‐assisted review required approximately 20 min, corresponding to a time reduction of around 30%. The time savings were primarily attributable to the automated evaluation of predefined metadata criteria, which reduced the need for manual inspection of individual results.

In addition to processing time, the results of the conventional operator‐based review were compared with those obtained using *MSVal*. In the 10 analytical runs evaluated, no questionable or non‐compliant results requiring exclusion were identified during the conventional review. *MSVal* correctly identified individual samples that did not meet predefined criteria for peak asymmetry, peak start/end time, and ion ratio. However, all of these samples had concentrations below the lower limit of quantification (LLOQ). Thus, the observed deviations in these metadata parameters were consistent with the low analyte concentrations and did not represent unexpected analytical findings.

For ivacaftor‐M1, *MSVal* also correctly identified samples above the LLOQ that did not meet the predefined ion ratio acceptance criterion (±20% of the calibrator ion ratio value). Review of the corresponding chromatograms showed that the qualifier ion peak had not been integrated correctly, resulting in an incorrect ion ratio calculation. In contrast, the quantifier ion peak was correctly integrated, and the quantitative result was therefore considered valid. Thus, *MSVal* correctly identified an issue with the ion ratio while the quantitative result itself remained unaffected. These findings demonstrate that *MSVal* reliably applies the predefined acceptance criteria and identifies results that do not meet individual criteria, while allowing the user to interpret these outputs in the context of the respective analytical method and sample concentration.

Another relevant method‐specific consideration is provided in the ‘Criteria Input’ field J30 of *MSVal*. This field allows the user to account for differences in RT between the analyte and its corresponding IS. This option is particularly relevant for the metabolites included in our method, ivacaftor‐M1, ivacaftor‐M6, tezacaftor‐M1 and elexacaftor‐M23, for which the RTs of the analyte and IS differ. This illustrates the flexibility of *MSVal* to accommodate method‐specific characteristics rather than relying on generic acceptance criteria. Additionally, *MSVal* can create a summary for longitudinal method monitoring from series to series with trend analyses regarding IS RT and IS area as well as matrix effects.

To further assess the ability of *MSVal* to identify non‐compliant results, an intentionally modified dataset was generated in which selected calibrator, QC, and sample results were altered to violate the predefined acceptance criteria. *MSVal* correctly identified these intentionally introduced failures and classified the respective results as not meeting the predefined criteria. This dataset is provided in the Supporting Information and allows the functionality of the tool to be independently assessed.

It should be noted that the acceptance criteria applied by *MSVal* are not intended to represent universal criteria for LC‐MS/MS methods. Rather, the criteria must be defined by the user according to the specific analytical method, analyte, and validation requirements. Each implementation of *MSVal* requires its own validation, meaning that the *MSVal*‐based validation process must be verified for the specific measurement method and the acceptance criteria individually specified in *MSVal*. In practice, this requires demonstrating that visually recognisable suspicious peak characteristics (e.g. co‐elution, incorrect peak integration, peak misassignment or inappropriate integration line) are reliably detected by the chosen *MSVal* criteria. Based on these findings, the *MSVal* workflow can be refined, including specifying which cases should be reviewed by the operator or a supervisor. Consequently, workflows using *MSVal* must be defined and validated locally by an end‐user.

The use‐case of this solution can be described as autovalidation, a term commonly used in the context of laboratory information systems (LIS). It refers to the automated identification of results and samples requiring particular review by an experienced analyst. To date, no comparable autovalidation solution has been published for LC‐MS/MS or other chromatographic methods. The term validation can, in principle, be defined as the act of declaring something to be valid. In area chromatographic–mass spectrometric analysis, the term is currently used predominantly to refer to the initial method validation performed prior to implementation in routine diagnostics. In the present work, however, we use the term validation in a broader sense, referring to the act of declaring an analytical run—or each individual sample within a series, respectively—to be valid for use in diagnostic procedures, provided that predefined acceptance criteria are met.

A software‐supported approach to result validation has the potential to enhance process safety. In practice, the enormous number of chromatograms generated during high‐throughput analyses involving multiple analytes poses a significant challenge when they are to be reviewed and reliably assessed by human experts alone—for example, in multi‐analyte steroid panels with several MRM transitions per analyte and per IS compound. Such measurement methods place considerable demand on highly trained personnel, and the repetitive nature of the task is both monotonous and fatiguing. As this requires a significant amount of working time from specialist staff who are becoming increasingly difficult to find, it also represents a considerable cost factor. We therefore believe that the type of tool described here can contribute to improved patient safety. Such systems should, however, be implemented and evaluated within a risk‐based framework, ensuring that risks associated with both software‐assisted and manual validation are evaluated and considered.

The *MSVal* system has been exemplarily implemented for MassLynx, an MS software package that has been in continuous use in our laboratory for more than 20 years. The *MSVal* macro, provided as Supporting Information, can be tested by the reader of this article accordingly. For software from other vendors, the output format (i.e. table column order) can be adapted as needed.

The downloadable MSVal file is provided to allow testing in the context of this publication; it is not intended and not approved to validate patient results in clinical practice. According to the EU‐IVDR, such a tool does not qualify as a medical device, as it merely checks data to support and improve the overall release of results by trained personnel in standard workflows. Similarly, LIS systems are not considered medical devices and are therefore not subject to certification requirements.

The solution presented here primarily demonstrates feasibility; a substantial development focus was on easy handling for the operators. It was not intended to support sophisticated primary data processing as described elsewhere [12, 13]. Several opportunities for further development can be considered, including integrating the recently described detuning ratio [14] and expanding longitudinal monitoring capabilities (e.g. peak‐width trends as indicators of chromatographic column performance). In this context, we aim to encourage commercial software providers to develop and offer comparable solutions, as there is a clear unmet need in this field and a gap in the global market. To our knowledge, no equivalent product is currently available outside the USA, where a respective software‐as‐a‐service solution is offered commercially [7].

Validated middleware for advanced mass spectrometric applications in clinical laboratories is consistent with broader standardisation initiatives addressing this inherently complex field [15]. In addition, such approaches aim to alleviate the considerable cognitive burden placed on laboratory personnel, as the complexity of these techniques and the requirement to process large volumes of data in a patient safety–critical setting may also represent a significant psychological burden.

*MSVal* was developed using conventional programming techniques within a widely used, generic software environment (Microsoft Excel), rather than based on machine learning [12]. This approach offers the advantage of full transparency. Future machine learning and AI‐based solutions may evolve into adaptive systems that learn from chromatograms and analytical runs accepted by human experts and subsequently apply these learned rules to new datasets. However, such approaches inherently reduce transparency and traceability. In contrast, the solution presented here is static, fully auditable, and entirely traceable. There is no doubt that other software frameworks, such as R, could also be used to develop a product like the one described here instead of Excel.

## 4 Conclusion

We have shown that middleware tools bridging MS chromatography software and LIS are feasible and hold great potential for improving analytical safety while reducing the burden on human resources. Assisted result release based on a clearly specified set of rules can thus free personnel from monotonous review tasks and redirect them to more productive activities. Further development of this approach through collaboration between software providers and potential end‐users toward commercially available products would be of great value, as it would address a genuine, as yet unmet need in laboratory diagnostics and other biomedical applications of quantitative MS.

## 5 Artificial Intelligence Use

Figure 1 was generated using Microsoft M365 Copilot and then reviewed and edited by the authors.

## Author Contributions

Conceptualisation: Verena Endt and Michael Vogeser. Methodology: Verena Endt, Katharina Habler and Michael Vogeser. Software: Verena Endt. Investigation: Verena Endt and Michael Vogeser. Data curation: Verena Endt, Katharina Habler and Michael Vogeser. Formal analysis: Verena Endt and Michael Vogeser. Validation: Verena Endt, Katharina Habler and Michael Vogeser. Project administration: Katharina Habler. Supervision: Katharina Habler and Michael Vogeser. Writing – original draft: Verena Endt and Michael Vogeser. Writing – review and editing: Katharina Habler.

## Ethics Statement

As only fully anonymised patient samples were used that were not obtained specifically for use in this study through an interaction or intervention with living individuals, neither informed consent nor IRB review was required.

## Conflicts of Interest

The authors declare no conflicts of interest.

## Funding

The authors have nothing to report.

## Supporting information




**Supporting File 1**: ansa70111‐sup‐0001‐SuppMat.xlsx.

**Supporting File 2**: ansa70111‐sup‐0002‐MSVal.xlsx.

## Data Availability

Data are provided via Supporting Information.
